# Effect of internal limiting membrane peeling on postoperative visual acuity in macula-off rhegmatogenous retinal detachment

**DOI:** 10.1371/journal.pone.0255827

**Published:** 2021-08-05

**Authors:** Shumpei Obata, Masashi Kakinoki, Osamu Sawada, Yoshitsugu Saishin, Yusuke Ichiyama, Masahito Ohji, on behalf of the Japan Retina Vitreous Society Registry Committee

**Affiliations:** Department of Ophthalmology, Shiga University of Medical Science, Shiga, Japan; Massachusetts Eye & Ear Infirmary, Harvard Medical School, UNITED STATES

## Abstract

**Purpose:**

To investigate the effects of internal limiting membrane (ILM) peeling on visual acuity (VA) after rhegmatogenous retinal detachment (RRD) surgery.

**Methods:**

This retrospective analysis examined the medical records of patients with RRD who underwent vitrectomy at 26 institutions. To detect prognostic factors of VA at 6 months postoperatively (post-VA), multivariate linear regression was performed with post-VA as the objective variable; ILM peeling, sex, age, preoperative VA (pre-VA), intraocular pressure, axial length, duration of RRD, and cataract surgery served as explanatory variables. Recurrence of RRD and epiretinal membrane formation within 6 months postoperatively were compared between groups of patients with and without ILM peeling, among patients with macula-on and macula-off RRD.

**Results:**

The inclusion criteria were met by 523 eyes with a macula-on RRD and 364 eyes with a macula-off RRD. ILM peeling was performed in 85 eyes with a macula-on RRD and 57 eyes with a macula-off RRD. In eyes with a macula-on RRD, ILM peeling did not affect post-VA (p = 0.72). Vitrectomy without cataract surgery and poor pre-VA were significantly associated with poor post-VA (p = 0.01 and p < 0.001, respectively). In eyes with a macula-off RRD, ILM peeling, long duration of RRD, and poor pre-VA were significantly associated with poor post-VA (p = 0.037, p = 0.007, and p < 0.001, respectively). Recurrence of RRD and epiretinal membrane formation were similar between groups of patients with and without ILM peeling, among patients with macula-on and macula-off RRD. Retina sensitivity was not evaluated by microperimetry.

**Conclusion:**

ILM peeling did not affect post-VA in eyes with a macula-on RRD, whereas post-VA was worse in eyes with ILM peeling than in eyes without peeling, among eyes with a macula-off RRD.

## Introduction

Vitrectomy is an effective treatment for rhegmatogenous retinal detachment (RRD), but an epiretinal membrane (ERM) may develop after surgery for RRD and secondary surgeries may be needed for ERM removal in some cases [1–4]. Internal limiting membrane (ILM) peeling during vitrectomy has been a common procedure for treating macular diseases such as macular hole or ERM. ILM peeling can effectively prevent recurrence of ERM after vitrectomy for ERM [5, 6]. ILM peeling during the primary surgery for RRD has also been reported to prevent recurrence of ERM [3, 7]. However, ILM peeling may cause retinal damage during the procedure and may slow recovery of vision [8–10]. Controversy remains regarding the effect of ILM peeling on visual outcomes following vitrectomy for RRD [2–4, 7, 11]. In this study, we investigate the effect of ILM peeling during primary vitrectomy for RRD on postoperative visual outcomes and ERM formation.

## Materials and methods

The committee of the Japan-Retinal Detachment Registry (J-RD Registry) was created in 2014. All members of the medical institutions of the Japan Retina Vitreous Society (JRVS) Committee who participated were screened to determine that their surgical techniques met the criteria set forth by the Committee. In these institutions, all surgeries were performed by specialists and qualified supervisors certified by the Japanese Ophthalmological Society. From February 2016 to March 2017, the medical records of patients who underwent surgery to treat RRD in 26 institutions, and who completed follow-up examinations for 6 months postoperatively, were registered through the internet; these data were retrospectively reviewed by the committee of the J-RD Registry. The data center was in the secretariat office of the JRVS, and the data were stored in that location and managed with the appropriate security [12]. The following data were derived from the J-RD Registry and analyzed: patient age, sex, preoperative visual acuity (VA), axial length, intraocular pressure, status of macular attachment, development of proliferative vitreoretinopathy (PVR), duration of retinal detachment (RD), ILM peeling, cataract surgery during the vitrectomy, tamponade substance, surgical adjuvant substance, use of an encircling band, recurrence of RD, postoperative VA and postoperative ERM formation. This study protocol was approved by the Institutional Review Board (IRB)/Ethics Committee Shiga University of Medical Science (Otsu, Japan). An opt-out consent process was used. This study adhered to the tenets of the Declaration of Helsinki.

Patients who had undergone vitrectomy for RRD, from among the patients in the registry, were included in this study. The exclusion criteria were: history of previous intraocular surgery, lack of information on macula-attachment status, absence of pre- and/or postoperative (6 months) VA and/or intraocular pressure and/or axial length and/or duration of RD, macular hole RD, RRD associated with atopic dermatitis, hereditary RRD, traumatic RRD, presence of macular disease, PVR or other disease that might contribute to visual impairment.

Statistical analyses were performed using GraphPad Prism 8 (GraphPad Software, Inc., La Jolla, CA). The best-corrected VA (BCVA) was measured using a Landolt C chart, and then converted to the logarithm of the minimum angle of resolution (logMAR) for analysis. The results are expressed as the mean ± standard deviation for continuous variables, and as proportions (%) for categorical variables. The Mann-Whitney U test was used for comparisons of independent groups. For comparisons of categorical data, Fisher exact tests were performed. In this study, the decision to perform, or not to perform, ILM peeling during RRD surgery was made by the surgeons. To eliminate the potential selection bias of surgeons performing ILM peeling in more-complicated cases, eyes with a history of ERM, uveitis or PVR, in which the surgeon is likely to perform ILM peeling, were excluded. Several factors have been reported to influence postoperative VA: preoperative VA, ILM peeling, duration of RD, high myopia, preoperative hypotony and PVR [2, 4, 13–16]. If cataract surgery is not performed during the RRD surgery, the cataract may progress postoperatively and postoperative VA may decrease [17]. Because the results from a non-randomized retrospective study may be affected by selection bias, multivariate linear regression was performed to evaluate the effect of ILM peeling on VA at 6 months postoperatively, by adjusting biases including selection bias. Because eyes with PVR were already excluded, multivariate linear regression analysis was performed with VA at 6 months postoperatively as the objective variable, and with ILM peeling, sex, age, preoperative VA, intraocular pressure, axial length, duration of RD and cataract surgery as the explanatory variables. The odds ratio and 95% confidence interval associated with each predictor were calculated from the linear regression models. Differences with p < 0.05 were considered statistically significant.

## Results

### Baseline characteristics

A total of 3446 eyes with RRD were registered as having been treated in the 26 hospitals from February 2016 to March 2017. Of those, 887 eyes with RRD in 25 hospitals met the above-mentioned inclusion/exclusion criteria. None of the eyes in one hospital met the inclusion/exclusion criteria. The macula was attached in 523 eyes at the time of vitrectomy, and the RD involved the macula in the other 364 eyes. All data are provided in S1 File. No statistically significant differences were found in the baseline characteristics in eyes with ILM peeling, versus without peeling, among patients with a macula-on RRD (Table 1). The duration of RRD was longer in the ILM peeling group than in the non-ILM peeling group in eyes with a macula-off RRD (Table 2). No statistically significant differences were found in the other baseline characteristics. Data on the use of an encircling band, cataract surgery, tamponade substance and surgical adjuvant substance are also shown in Tables 1 and 2. The proportion of patients who underwent cataract surgery in the ILM peeling group was significantly higher than that in the non-ILM peeling group among those with a macula-on RRD (p = 0.005).

**Table 1:** Baseline characteristics and surgical details for the eyes with a macula-on rhegmatogenous retinal detachment.

	With ILM^a^ peeling (85 eyes)	Without ILM peeling (438 eyes)	P-value
Sex (male:female)	48: 37	285: 153	0.14
Age (years)	59.2 ± 8.0	58.0 ± 9.4	0.16
Axial length (mm)	25.3 ± 1.6	25.5 ± 2.1	0.26
logMAR BCVA^b^	0.16 ± 0.51	0.16 ± 0.56	0.57
Intraocular pressure (mm Hg)	13.3 ± 3.0	13.7 ± 3.2	0.24
Duration of RD^c^ (days)	15.1 ± 20	13.6 ± 29	0.64
Use of encircling band (eyes, %)	1 (1.2%)	20 (4.6%)	0.23
Cataract surgery (eyes, %)	79 (92.9%)	353 (80.6%)	0.005
Tamponade substance (eyes)	14: 69: 0: 2	105: 321: 3: 8	0.39
Air: SF_6_^d^: C_3_F_8_^e^: SO^f^			
Surgical adjuvant substance (eyes)	73: 53: 9: 0	366: 2: 0: 1	< 0.001
TA^g^: BBG^h^: ICG^i^: TB^j^			
Use of PFCL^k^ (eyes)	4	32	0.39

^a^ ILM: Internal limiting membrane.

^b^ logMAR BCVA: Logarithm of the minimum angle of resolution best-corrected visual acuity.

^c^ RD: Retinal detachment.

^d^ SF6: Sulfur hexafluoride.

^e^ C3F8: Perfluoropropane.

^f^ SO: Silicone oil.

^g^ TA: Triamcinolone acetonide.

^h^ BBG: Brilliant Blue G.

^I^ ICG: Indocyanine green.

^j^ TB: Trypan blue.

^k^ PFCL: Perfluorocarbon liquids.

**Table 2:** Baseline characteristics and surgical details for the eyes with a macula-off rhegmatogenous retinal detachment.

	With ILM^a^ peeling (57 eyes)	Without ILM peeling (307 eyes)	P-value
Sex (male:female)	43: 14	214: 93	0.43
Age (years)	59.5 ± 9.4	59.3 ± 9.4	0.82
Axial length (mm)	25.2 ± 2.0	25.3 ± 1.7	0.84
logMAR BCVA^b^	0.94 ± 0.65	1.1 ± 0.77	0.38
Intraocular pressure (mm Hg)	12.4 ± 3.4	12.8 ± 3.4	0.55
Duration of RD^c^ (days)	15.5 ± 15	13.1 ± 29	0.02
Use of encircling band (eyes, %)	0 (0%)	10 (3.3%)	0.37
Cataract surgery (eyes, %)	54 (94.7%)	261 (85.0%)	0.06
Tamponade substance (eyes)	10: 46: 1: 0	43: 248: 4: 11	0.47
Air: SF_6_^d^: C_3_F_8_^e^: SO^f^			
Surgical adjuvant substance (eyes)	55: 35: 8	269: 2: 0	< 0.001
TA^g^: BBG^h^: ICG^i^:			
Use of PFCL^j^ (eyes)	4	43	0.15

^a^ ILM: Internal limiting membrane.

^b^ logMAR BCVA: Logarithm of the minimum angle of resolution best-corrected visual acuity.

^c^ RD: Retinal detachment.

^d^ SF6: Sulfur hexafluoride.

^e^ C3F8: Perfluoropropane.

^f^ SO: Silicone oil.

^**g**^ TA: Triamcinolone acetonide.

^h^ BBG: Brilliant Blue G.

^i^ ICG: Indocyanine green.

^j^ PFCL: Perfluorocarbon liquids.

### Factors affecting postoperative VA

In the eyes with a macula-on RRD, 6-month postoperative VA was not significantly different between eyes with ILM peeling and those without ILM peeling (−0.01 and −0.02 logMAR, respectively, p = 0.08). Multivariate linear regression analysis, performed using the data from 85 eyes with ILM peeling and 438 eyes without ILM peeling, showed that ILM peeling did not affect the postoperative VA at 6 months (β value = 0.009, p = 0.72). Vitrectomy without cataract surgery in phakic eyes and poor preoperative VA were significantly associated with poor VA at 6 months after surgery (Table 3).

**Table 3:** Multiple linear regression analysis for VA at 6 months postoperatively in the eyes with a macula-on Rhegmatogenous Retinal Detachment (RRD).

	β value	Standard error	95% confidence interval	P-value
Without internal limiting membrane peeling	−0.009	0.027	−0.062 to 0.043	0.72
Sex (male)	−0.007	0.021	−0.047 to 0.033	0.73
Age	0.002	0.0013	−0.0004 to 0.005	0.10
Preoperative visual acuity	0.09	0.018	0.058 to 0.13	<0.001
Intraocular pressure	−0.006	0.0032	−0.012 to 0.0007	0.08
Axial length	−0.00006	0.00096	−0.0003 to 0.0001	0.49
Duration of RRD	−0.0002	0.029	−0.0009 to 0.0005	0.60
Without cataract surgery	0.07	0.00035	0.017 to 0.13	0.01

In the eyes with a macula-off RRD, 6-month postoperative VA in the ILM peeling group was significantly worse than that in the non-ILM peeling group (0.20 and 0.14 logMAR, respectively, p = 0.02). ILM peeling was significantly associated with poor VA at 6 months post-vitrectomy in the analysis by multiple linear regression, after adjusting for the baseline characteristics (β = −0.075, p = 0.037). A long duration of RRD and poor preoperative VA were also significantly associated with poorer VA at 6 months after surgery (Table 4).

**Table 4:** Multiple linear regression analysis for VA at 6 months postoperatively in the eyes with a macula-off Rhegmatogenous Retinal Detachment (RRD).

	β value	Standard error	95% confidence interval	P-value
Without internal limiting membrane peeling	−0.075	0.036	−0.15 to -0.0046	0.037
Sex (male)	-0.036	0.029	−0.09 to 0.02	0.22
Age	0.003	0.0017	−0.00006 to 0.007	0.054
Preoperative visual acuity	0.10	0.018	0.066 to 0.14	<0.001
Intraocular pressure	−0.007	0.0039	−0.014 to 0.001	0.09
Axial length	−0.0017	0.0091	−0.02 to 0.016	0.85
Duration of RRD	0.0013	0.00049	0.00036 to 0.0023	0.007
Without cataract surgery	0.06	0.041	-0.02 to 0.14	0.14

### ERM formation

In the eyes with a macula-on RRD, an ERM developed in 3 eyes (3.5%) with ILM peeling and in 30 eyes (6.9%) without ILM peeling (p = 0.33). Surgery for an ERM was not needed in any eyes with ILM peeling; it was needed in 11 eyes (2.5%) without ILM peeling (p = 0.23,).

In the eyes with a macula-off RRD, an ERM developed in 2 eyes (3.5%) with ILM peeling and in 24 eyes (7.8%) without ILM peeling (p = 0.40). Surgery for ERM was not needed in the eyes with ILM peeling; it was needed in 4 eyes (1.3%) without ILM peeling (p > 0.99).

### Recurrence of RRD

In the eyes with a macula-on RRD, 2 eyes (2.4%) in the ILM peeling group and 13 eyes (3.0%) in the non-ILM peeling group required secondary surgery to address recurrence of the RRD (p > 0.99, Fisher exact test).

In the eyes with a macula-off RRD, 3 eyes (5.3%) in the ILM peeling group and 11 eyes (3.6%) in the non-ILM peeling group needed secondary surgery for recurrence of RRD (p = 0.47, Fisher exact test).

## Discussion

We compared functional and anatomic changes after vitrectomy for RRD. In the eyes with a macula-on RRD, ILM peeling did not affect VA at 6 months postoperatively, after adjusting for baseline characteristics. However, poor preoperative VA and vitrectomy without cataract surgery were significantly associated with poor VA at 6 months. Conversely, in the macula-off RRD group, ILM peeling was significantly associated with poor VA at 6 months postoperatively. A long duration of RD and poor preoperative VA also were significantly associated with poor VA at 6 months, after adjusting for baseline characteristics, as in a previous report [16]. Surgeons are not able to choose and/or change some risk factors, such as preoperative VA. However, ILM peeling, cataract surgery during the RRD surgery and time from the onset of RD to surgery are manageable factors, at the surgeon’s discretion.

There have been several previous reports investigating whether or not ILM peeling affects VA after surgery for RRD. However, some reports had only a small number of cases and multiple linear regression analysis was rarely used because of the small sample sizes. In the current study, we investigated whether it is advisable to perform ILM peeling during RRD surgery, using multiple linear regression analysis with this much-larger number of cases.

Nam et al. [2] reported that postoperative VA was better in eyes in their ILM peeling group than in eyes without ILM peeling, in eyes with a macula-on RRD. They postulated that ILM peeling achieved better VA by preventing the development of postoperative pucker. In the current study, however, ILM peeling did not affect VA at 6 months postoperatively in the macula-on group, after adjusting for baseline characteristics.

ERM formation is a common complication after RRD surgery. ILM peeling has been proposed to prevent ERM formation in eyes after vitrectomy [2, 3, 18]. Nam et al. [2] reported that an ERM developed in 21.5% of eyes after vitrectomy without ILM peeling whereas it developed in only 6.4% of eyes with ILM peeling. In the current study, there were fewer eyes with ERM formation after RRD surgery in the ILM peeling group than in the non-ILM peeling group, but the difference was not statistically significant (p = 0.33). This distinction between our study and the report by Nam et al. might arise from the differences in duration of RD, follow-up period and definition of ERM.

In eyes with a macula-off RRD, Eissa et al. [4] reported that ILM peeling caused adverse effects on the retina and resulted in poor postoperative VA. This agrees with the results in the current study, which had many more cases. In the current study, ILM peeling was significantly associated with poor VA at 6 months in the analysis using multiple linear regression, adjusted for baseline characteristics (age, sex, intraocular pressure, axial length, vitrectomy with or without cataract surgery, preoperative VA and duration of RD) (β = −0.075, p = 0.037, multiple linear regression). The mechanism underlying poor VA associated with ILM peeling may be retinal damage caused by the ILM peeling procedure. Dissociated optic nerve fiber layer (DONFL) has been reported to occur in eyes with ILM peeling. DONFL is thought to develop because of the loss of Muller cells, which might result in lower retinal sensitivity postoperatively and poor VA [8, 19–23]. ILM peeling from a detached retina is more difficult than that from an attached retina. This may cause greater damage to the retina in eyes with a macula-off RRD.

Simultaneous cataract surgery during vitrectomy for macula-on RRD was associated with better VA at 6 months postoperatively in the current study. Nuclear sclerotic cataracts progress after a vitrectomy without cataract surgery in phakic eyes, especially in patients more than 50 years old [17]. Simultaneous cataract surgery also allows surgeons to remove far-peripheral vitreous safely and more completely. Simultaneous cataract surgery during vitrectomy for RRD should be considered in patients older than 50. Simultaneous cataract surgery may be spared in patients younger than 50 because they have accommodation ability and because progression of nuclear cataract is less common in younger patients [17].

Our results showed that a longer duration of RD in eyes with a macula-off RRD was significantly associated with poor VA at 6 months. This agrees with previous studies in which poorer visual outcomes were associated with longer durations of preoperative macular detachment [24, 25]. Kim et al. reported that surgical repair within 6 days of symptom onset yielded better visual outcomes than did surgeries performed later, and that visual outcome was not affected by the timing of the surgical repair after 7 days [26]. Surgery should be performed as soon as possible in eyes with a macula-off RRD because of the effect on postoperative VA.

The current study has several limitations. First, this study was retrospective and nonrandomized, which could have introduced selection bias. The decision to perform, or not to perform, ILM peeling during RRD surgery was made by the surgeons, based on their own preference; we could not completely eliminate the potential selection bias of surgeons to perform ILM peeling in more-complicated cases. To address that concern, eyes with a history of ERM, uveitis or PVR, in which the surgeon is likely to perform ILM peeling, were excluded. Almost all baseline characteristics were similar between eyes with ILM peeling and those without. Therefore, selection bias seems to be minimal. Moreover, multiple regression analysis was performed to exclude or minimize the selection bias as much as possible. Even after the multiple regression analysis, ILM peeling was a risk factor for poor post-operative VA in the eyes with a macula-off RRD. Second, the follow-up period was 6 months; results with longer follow-up periods would be preferable. Because this was a multicenter study, it was almost impossible to obtain follow-up data for a longer period than that used for data collection for the Registry. However, the follow-up period was also at least 6 months after the initial vitrectomy in other reports [3, 27]. Those reports did not include follow-up for a full 12 months. Moreover, Sousa et al. found no difference in postoperative VA between 6-month and 12-month follow-up [13]. Therefore, VA measurement at 6 months after surgery was considered acceptable. A strength of the current study is the inclusion of data from a large number of eyes, from 25 hospitals, compared with similar reports in the literature. Data from multiple hospitals may support a more-universal conclusion. Finally, the evaluation of microperimetry lacked and so the sensitivity of the retina was not evaluated.

In conclusion, ILM peeling did not affect VA at 6 months postoperatively in eyes with a macula-on RRD, whereas VA at 6 months postoperatively was worse in eyes with ILM peeling than in eyes without ILM peeling in the eyes with a macula-off RRD. This suggests that it is not necessary to peel the ILM during RRD surgery in eyes with a macula-on RRD and that ILM peeling should not be performed in eyes with a macula-off RRD because of the adverse effect on postoperative VA.

## Supporting information

S1 File Data set for analysis. (XLSX)
